# Gastric Perforation in a Patient Receiving Neoadjuvant Chemoradiotherapy

**DOI:** 10.14740/wjon924w

**Published:** 2015-06-12

**Authors:** Hui Chen, Susan Wu, Ajay Kundra, Iheanyichukwu Aja Onu, Vladimir Gotlieb, Jen C. Wang

**Affiliations:** a Division of Hematology/Oncology, Brookdale University Hospital Medical Center, Brooklyn, NY, USA; b Department of Pathology, South Nassau Communities Hospital, Oceanside, NY, USA; c Department of Gastroenterology, South Nassau Communities Hospital, Oceanside, NY, USA

**Keywords:** Gastrointestinal perforation, Gastric cancer, Chemoradiotherapy

## Abstract

Perioperative chemoradiotherapy is considered to be one of the standards of care for early-stage gastric cancer, especially when it involves the esophagogastric junction or greater curvature. To date, there are no reported cases of gastrointestinal perforation in the literature, including many major clinical trials of adjuvant or neoadjuvant chemoradiotherapy for gastric cancer. It is important to recognize and manage this rare, but fatal complication in a timely manner. We report one case of gastrointestinal perforation in a gastric cancer patient undergoing neoadjuvant chemoradiotherapy with 5-fluorouracil and oxaliplatin. A 75-year-old man was diagnosed with stage IV gastric cancer (T4N1M0). We started neoadjuvant chemoradiotherapy with 5-fluorouracil and oxaliplatin. After he finished the first cycle, the patient presented to emergency room with severe abdominal pain of sudden onset. Computed tomography showed moderate pneumoperitoneum and perihepatic fluid. The patient expired 6 hours after he presented to emergency room.

## Introduction

Gastric cancer is the fourth most frequently diagnosed cancer, and the second leading cause of death from cancer worldwide [1]. In most parts of the world, gastric cancer is often diagnosed at an advanced stage and continues to pose a major challenge for healthcare professionals.

In multiple clinical trials, perioperative chemoradiotherapy has improved survival among patients with potentially curable gastric cancer [2-9]. Chemoradiotherapy may induce febrile neutropenia and gastrointestinal bleeding. Gastric perforation appears to be a very rare occurrence; we have reviewed the literature, including the major clinical trials for perioperative chemoradiotherapy for gastric cancer, and did not find any reported cases [2-9]. Herein, we report one patient with gastric cancer in whom spontaneous gastrointestinal perforation occurred during neoadjuvant chemoradiotherapy with 5-fluorouracil and oxaliplatin. It is critical to recognize this rare but life-threatening complication and manage it in a timely manner.

## Case Report

A75-year-old man presented to the emergency room on January 02, 2015 with chief complaint of abdominal pain for 3 weeks, associated with nausea and vomiting, and weight loss of about 15 pounds. He denied any change in his bowel habits, and there was no history of overt gastrointestinal bleeding. He had a colonoscopy 2 years prior, but never had an upper endoscopy. There was no family history of gastrointestinal neoplasia.

**Figure 1:**
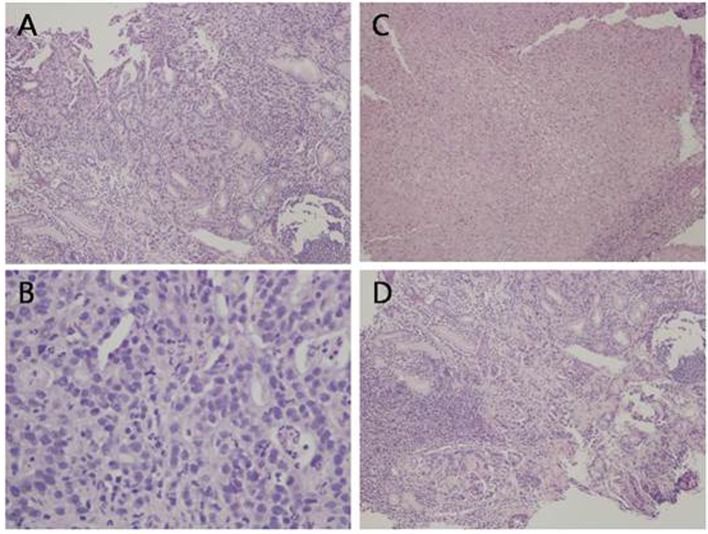
Histological analysis of the gastric ulcer biopsy showing malignant glandular proliferation (A, H&E, × 10), tumor cells exhibiting high nuclear to cytoplasmic ratio, prominent nucleoli, and frequent mitotic figures (B, H&E, × 40). There are areas of ulceration with tissue necrosis (C, H&E, × 10) and areas of lymphocytic infiltrate, morphologically consistent with reactive process (D, H&E, × 10).

Computed tomography (CT) of the abdomen and pelvis revealed thickening of the gastric fundus with lymph nodes measuring up to 1.2 cm in the gastrohepatic ligament and mild hazy soft tissue in the omentum on the left side of the abdomen. Upper endoscopy and biopsy was then performed. Along the lesser curvature of the stomach, extending from the incisura angularis to the cardia, a large ulcer crater was noted. The base of the ulcer showed granulation tissue without visible vessels, no active bleeding, and the antrum showed mild erythema. Pathology of the ulcer biopsy showed invasive adenocarcinoma, moderately differentiated with ulceration and necrosis (Fig. 1). Gastric antrum biopsy showed mild to moderate chronic active gastritis and extensive intestinal metaplasia, negative for dysplasia. *Helicobacter pylori* were not seen on hematoxylin-eosin stain and immunostain. CT scan of the chest was done and there was no evidence of pulmonary metastasis.

The patient was started the neoadjuvant chemoradiotherapy treatment with 5-fluorouracil and oxaliplatin using the following regimen as recommended in NCCN guideline [10]: oxaliplatin 85 mg/m^2^ IV on day 1, leucovorin 400 mg/m^2^ IV on day 1, 5-fluorouracil 400 mg/m^2^ IV push on day 1, 5-fluorouracil 800 mg/m^2^ IV continuous infusion over 24 h daily on days 1 and 2. The regimen is cycled every 14 days for three cycles with radiation and three cycles after radiation. The patient tolerated the chemoradiotherapy very well and during the first week of treatment, his appetite improved. The symptoms of abdominal pain, nausea and vomiting resolved.

**Figure 2:**
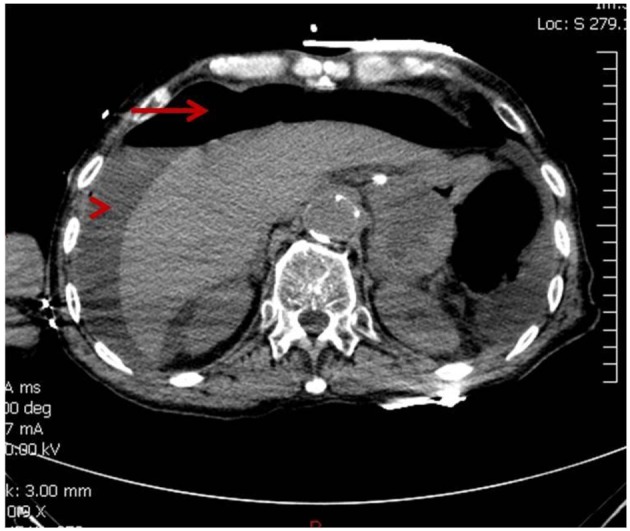
CT showing moderate pneumoperitoneum (arrow) and perihepatic fluid (arrow head).

At the beginning of the second cycle, the patient came to the hospital for radiation and complained of severe abdominal pain but was sent home with analgesics. The next day he came back to the emergency room with persistent severe abdominal pain, accompanied with decreased appetite and diarrhea. Abdominal X-ray showed questionable free air in the abdomen, so a non-contrast CT scan was performed to clarify. CT scan revealed the development of moderate pneumoperitoneum and perihepatic fluid (Fig. 2). Patient went into cardiac arrest and was resuscitated, but required ventilator support. Due to the overall critical condition, no surgical intervention was performed. He expired 6 h after he arrived at the emergency room.

## Discussion

Spontaneous perforation is a rare complication of gastric cancer. The reported incidence of perforation of gastric cancer is less than 5% [11-15]. Amongst these patients, the majority have advanced disease, with 64-88% presenting with stage III/IV disease [14, 15]. Perforation of gastrointestinal tract during treatment is even more rare, and in the major clinical trials for perioperative chemotherapy or chemoradiotherapy, perforation of gastrointestinal tract was not listed among the adverse effects [2-9]. For example, in the MAGIC trial, a total of 553 patients were studied, 250 of which received perioperative chemotherapy, and no incidence of gastrointestinal perforation was reported in this trial [7]. In the SWOG-9008 trial of the adjuvant chemoradiotherapy for adenocarcinoma of the stomach or esophagogastric junction, gastrointestinal perforation was not reported as one of the adverse effects in the 556 cases, 281 of which received chemoradiotherapy [3].

There was one case of esophageal perforation in the CROSS trial [16], in which neoadjuvant chemoradiotherapy with carboplatin and paclitaxel was studied on esophageal or esophagogastric-junction cancer. However, there were no occurrences of gastric perforation in this study. In a clinical trial using neoadjuvant chemoradiotherapy with carboplatin and paclitaxel for locally advanced gastric cancer, there was no reported incidence of perforation during chemoradiotherapy in the 25 cases, although there were two cases of bowel perforation as surgery-related complication [17]. In the phase I study of neoadjuvant chemoradiotherapy with S-1 and oxaliplatin in 12 patients with locally advanced gastric cancer, no gastrointestinal perforation was reported [18]. Outside major clinical trials, there are very few case reports of gastrointestinal perforation during chemoradiotherapy in the metastatic setting, so far only two cases reported during chemotherapy with docetaxel and S-1 [19, 20].

The gastrointestinal perforation in our patient can be due to the following causes. The first is spontaneous tumor rupture due to the progress of the ulcerative mass. The second is rapid tumor shrinkage and necrosis due to chemotherapy and radiotherapy. The third is direct toxicity of the drugs, such as chemotherapy agents or corticosteroids. In the aforementioned clinical trials, Borrmann’s classification of gastric carcinoma was not described. In general, the ulcerating carcinoma (type III) represents 25% of all advanced gastric carcinomas [21]. Even though ulcerating carcinoma is a common type of advanced gastric carcinoma, incidence of spontaneous gastrointestinal perforation in gastric cancer remains very low. Therefore, ulceration per se is not the major attributable factor to perforation. Our patient was treated with 5-fluorouracil and oxaliplatin. 5-fluorouracil is toxic to the gastrointestinal mucosa and induces gastroduodenal ulceration, gastritis, and duodenitis [22]. S-1 is an oral 5-fluorouracil, and gastrointestinal perforation was previously reported in chemotherapy with docetaxel and S-1 for gastric carcinoma [19, 20]. It is likely that the chemoradiotherapy played a role in the pathogenesis of perforation, due to the rapid tumor shrinkage and necrosis and the direct toxicity of the drug.

In multiple clinical trials, perioperative chemoradiotherapy has improved survival among patients with potentially curable gastric cancer [2-9]. As perioperative chemoradiotherapy has become the standard of care for early-stage gastric cancer and is widely used, it is important to recognize gastrointestinal perforation as a rare but fatal complication. Perforations that occur during chemotherapy are life-threatening because of immunosuppression. Perforation of gastric carcinoma results in peritonitis and acute abdominal syndrome, which can lead to multi-organ failure and death. Surgery or drainage should be performed in a timely manner for suitable patients.

In summary, gastrointestinal perforation is a rare but devastating complication of gastric cancer. Clinicians should be vigilant to this complication when they encounter gastric cancer patients with severe abdominal pain of sudden onset.

## References

[1] Jemal A, Bray F, Center MM, Ferlay J, Ward E, Forman D (2011). Global cancer statistics. CA Cancer J Clin.

[2] Lowy AM, Feig BW, Janjan N, Rich TA, Pisters PW, Ajani JA, Mansfield PF (2001). A pilot study of preoperative chemoradiotherapy for resectable gastric cancer. Ann Surg Oncol.

[3] Macdonald JS, Smalley SR, Benedetti J, Hundahl SA, Estes NC, Stemmermann GN, Haller DG (2001). Chemoradiotherapy after surgery compared with surgery alone for adenocarcinoma of the stomach or gastroesophageal junction. N Engl J Med.

[4] Ajani JA, Mansfield PF, Janjan N, Morris J, Pisters PW, Lynch PM, Feig B (2004). Multi-institutional trial of preoperative chemoradiotherapy in patients with potentially resectable gastric carcinoma. J Clin Oncol.

[5] Ajani JA, Mansfield PF, Crane CH, Wu TT, Lunagomez S, Lynch PM, Janjan N (2005). Paclitaxel-based chemoradiotherapy in localized gastric carcinoma: degree of pathologic response and not clinical parameters dictated patient outcome. J Clin Oncol.

[6] Ajani JA, Winter K, Okawara GS, Donohue JH, Pisters PW, Crane CH, Greskovich JF (2006). Phase II trial of preoperative chemoradiation in patients with localized gastric adenocarcinoma (RTOG 9904): quality of combined modality therapy and pathologic response. J Clin Oncol.

[7] Cunningham D, Allum WH, Stenning SP, Thompson JN, Van de Velde CJ, Nicolson M, Scarffe JH (2006). Perioperative chemotherapy versus surgery alone for resectable gastroesophageal cancer. N Engl J Med.

[8] Rivera F, Galan M, Tabernero J, Cervantes A, Vega-Villegas ME, Gallego J, Laquente B (2009). Phase II trial of preoperative irinotecan-cisplatin followed by concurrent irinotecan-cisplatin and radiotherapy for resectable locally advanced gastric and esophagogastric junction adenocarcinoma. Int J Radiat Oncol Biol Phys.

[9] Smalley SR, Benedetti JK, Haller DG, Hundahl SA, Estes NC, Ajani JA, Gunderson LL (2012). Updated analysis of SWOG-directed intergroup study 0116: a phase III trial of adjuvant radiochemotherapy versus observation after curative gastric cancer resection. J Clin Oncol.

[10] Conroy T, Galais MP, Raoul JL, Bouche O, Gourgou-Bourgade S, Douillard JY, Etienne PL (2014). Definitive chemoradiotherapy with FOLFOX versus fluorouracil and cisplatin in patients with oesophageal cancer (PRODIGE5/ACCORD17): final results of a randomised, phase 2/3 trial. Lancet Oncol.

[11] Gertsch P, Yip SK, Chow LW, Lauder IJ (1995). Free perforation of gastric carcinoma. Results of surgical treatment. Arch Surg.

[12] Adachi Y, Mori M, Maehara Y, Matsumata T, Okudaira Y, Sugimachi K (1997). Surgical results of perforated gastric carcinoma: an analysis of 155 Japanese patients. Am J Gastroenterol.

[13] Kitakado Y, Tanigawa N, Muraoka R (1997). [A case report of perforated early gastric cancer]. Nihon Geka Hokan.

[14] Roviello F, Rossi S, Marrelli D, De Manzoni G, Pedrazzani C, Morgagni P, Corso G (2006). Perforated gastric carcinoma: a report of 10 cases and review of the literature. World J Surg Oncol.

[15] Mahar AL, Brar SS, Coburn NG, Law C, Helyer LK (2012). Surgical management of gastric perforation in the setting of gastric cancer. Gastric Cancer.

[16] van Hagen P, Hulshof MC, van Lanschot JJ, Steyerberg EW, van Berge Henegouwen MI, Wijnhoven BP, Richel DJ (2012). Preoperative chemoradiotherapy for esophageal or junctional cancer. N Engl J Med.

[17] Trip AK, Poppema BJ, van Berge Henegouwen MI, Siemerink E, Beukema JC, Verheij M, Plukker JT (2014). Preoperative chemoradiotherapy in locally advanced gastric cancer, a phase I/II feasibility and efficacy study. Radiother Oncol.

[18] Lee DJ, Sohn TS, Lim do H, Ahn HK, Park SH, Lee J, Park JO (2012). Phase I study of neoadjuvant chemoradiotherapy with S-1 and oxaliplatin in patients with locally advanced gastric cancer. Cancer Chemother Pharmacol.

[19] Park SR, Kim HK, Kim CG, Choi IJ, Lee JS, Lee JH, Ryu KW (2008). Phase I/II study of S-1 combined with weekly docetaxel in patients with metastatic gastric carcinoma. Br J Cancer.

[20] Yamada T, Kanazawa Y, Yokoi K, Uchida E (2013). A case of gastric cancer with perforation caused by chemotherapy with docetaxel and S-1. J Nippon Med Sch.

[21] Lauwers GY, Odze RD, Goldblum JR (2009). Epithelial neoplasms of the stomach. Surgical Pathology of the GI Tract, Liver, Biliary Tract, and Pancreas.

[22] Grem JL, Chabner BA, Collins JM (1990). Fluorinated pyrimidines. Cancerchemotherapy: principles and practice.

